# FROM COMPLEX EVOLVING TO SIMPLE: CURRENT REVISIONAL AND ENDOSCOPIC PROCEDURES FOLLOWING BARIATRIC SURGERY

**DOI:** 10.1590/0102-6720201600S10031

**Published:** 2016

**Authors:** Ricardo ZORRON, Manoel Passos GALVÃO-NETO, Josemberg CAMPOS, Alcides José BRANCO, José SAMPAIO, Tido JUNGHANS, Claudia BOTHE, Christian BENZING, Felix KRENZIEN

**Affiliations:** 1 Center for Innovative Surgery (ZIC), Department of General, Visceral and Transplant Surgery, Campus Virchow Klinikum and Department of General, Visceral, Vascular and Thoracic Surgery, Campus Mitte, Charité-Universitätsmedizin Berlin, Berlin, Germany;; 2 Department of Bariatric Endoscopy, Gastrobeso Center, São Paulo, SP, Brazil;; 3 Department of Surgery, University Federal of Pernambuco, Recife, PE, Brazil;; 4 Department of Surgery, CEVIP Center, Curitiba, PR, Brazil; 5 Department for General, Visceral, Thorax and Vascular Surgery, Klinikum Bremerhaven Reinkenheide, Bremerhaven, Germany

**Keywords:** Bariatric surgery, Obesity, Metabolic surgery, Operative technique, Procedure selection, Gastric bypass, Gastric banding, Endoscopy

## Abstract

**Background:**

Roux-en-Y gastric bypass (RYGB) is a standard therapy in bariatric surgery. Sleeve gastrectomy and gastric banding, although with good results in the literature, are showing higher rates of treatment failure to reduce obesity-associated morbidity and body weight. Other problems after bariatric may occur, as band erosion, gastroesophageal reflux disease and might be refractory to medication. Therefore, a laparoscopic conversion to a RYGB can be an effective alternative, as long as specific indications for revision are fulfilled.

**Objective:**

The objective of this study was to analyse own and literature data on revisional bariatric procedures to evaluate best alternatives to current practice.

**Methods:**

Institutional experience and systematic review from the literature on revisional bariatric surgery.

**Results:**

Endoscopic procedures are recently applied to ameliorate failure and complications of bariatric procedures. Therapy failure following RYGB occurs in up to 20%. Transoral outlet reduction is currently an alternative method to reduce the gastrojejunal anastomosis. The diameter and volume of sleeve gastrectomy can enlarge as well, which can be reduced by endoscopic full-thickness sutures longitudinally. Dumping syndrome and severe hypoglycemic episodes (neuroglycopenia) can be present in patients following RYGB. The hypoglycemic episodes have to be evaluated and usually can be treated conventionally. To avoid partial pancreatectomy or conversion to normal anatomy, a new laparoscopic approach with remnant gastric resection and jejunal interposition can be applied in non-responders alternatively. Hypoglycemic episodes are ameliorated while weight loss is sustained.

**Conclusion:**

Revisional and endoscopic procedures following bariatric surgery in patients with collateral symptomatic or treatment failure can be applied. Conventional non-surgical approaches should have been applied intensively before a revisional surgery will be indicated. Former complex surgical revisional procedures are evolving to less complicated endoscopic solutions.

## RESUMO

**Racional:**

Bypass gástrico em Y-de-Roux (BGYR) é procedimento padrão em cirurgia bariátrica. Gastrectomia vertical e banda gástrica, embora com bons resultados na literatura, estão mostrando taxas mais elevadas de insucesso no tratamento para reduzir a morbidade associada à obesidade e peso corporal. Outros problemas pós-operatórios podem ocorrer, como a erosão da banda, e doença do refluxo gastroesofágico refratária à medicação. Portanto, conversão laparoscópica para BGYR pode ser alternativa eficaz, desde que indicações específicas para a revisão sejam cumpridas.

**Objetivo:**

Analisar os nossos dados e os da literatura sobre procedimentos bariátricos revisionais para avaliar melhores alternativas para a prática atual.

**Métodos:**

Foram efetuados experiência institucional e revisão sistemática da literatura sobre cirurgia bariátrica revisional.

**Resultados:**

Procedimentos endoscópicos estão sendo aplicados recentemente para melhorar a falha e complicações de procedimentos bariátricos. Falha terapêutica após BGYR ocorre em até 20%. A redução transoral é atualmente um método alternativo para reduzir a anastomose gastrojejunal. A gastrectomia vertical pode apresentar aumento de volume e do diâmetro do *pouch* , o qual podem ser reduzidos por meio de sutura total endoscópica longitudinal. Síndrome de dumping e episódios de hipoglicemia grave (neuroglicopenia) podem estar presentes nos pacientes com BGYR. Os episódios hipoglicêmicos devem ser avaliados e geralmente podem ser tratados convencionalmente. Para evitar pancreatectomia parcial ou conversão à anatomia normal, uma nova abordagem laparoscópica com ressecção do remanescente gástrico e interposição de jejuno, pode ser aplicada como alternativa em não-respondedores. Episódios de hipoglicemia melhoram, enquanto a perda de peso é mantida.

**Conclusão:**

Procedimentos revisionais endoscópicos podem ser aplicados após cirurgia bariátrica em pacientes com sintomas colaterais ou na falha do tratamento. Abordagens convencionais não-cirúrgicas devem ser aplicadas intensivamente antes que uma operação revisional seja indicada. Antigos procedimentos cirúrgicos revisionais complexos estão evoluindo para soluções endoscópicas menos complicadas.

## INTRODUCTION

Morbid obesity and related comorbidities are becoming increasingly important for the health system with growing incidence and prevalence, particularly in the Western nations. According to the World Health Organization, more than 1.9 billion people are overweight (2014), of which 600 million people are obese (body mass index BMI>30 kg /m^2)^ ^1^. Obesity is a major risk factor for diabetes, cardiovascular disease and thus has enormous consequences for the health system itself.

Bariatric and metabolic surgical procedures are superior compared to conservative multimodal therapies for morbid obesity^2^ ^,^ ^3^. For example, type 2 diabetes mellitus, hypertension, dyslipidemia and sleep apnea syndrome are successfully treated in most cases^4^. This has led to the acceptance of bariatric surgery, which has increased rapidly worldwide in the last 20 years. In 2003 approximately 150.000 bariatric procedures were performed, and in 2013 already it turned to be around 470.000 interventions^5^. The success of bariatric surgery is defined in terms of reduction in obesity-associated morbidity and a successful weight reduction^6^. Roux-en-Y gastric bypass (RYGB) is the gold standard and the most commonly performed bariatric surgery with a relative proportion of approximately 45%^5^, although laparoscopic sleeve gastrectomy (LSG) just have gained the position of most performed bariatric procedure in many countries.

The results of bariatric surgery are convincing, although some of the patients have a regain in body weight or the achieved weight loss is insufficient. The etiology of such so-called treatment failure is multifactorial and may causally be patient-dependent (nutrition, metabolism, hormonal status, physical activity) and technical-dependent factors (complications, type of procedure)^7^. The weight regain in initial normal course is typically associated with the recurrence of the comorbidities.

The aim of this article was to review and analyze the technical aspects of bariatric conversion procedures and endoscopic revision procedures introduced which can be used in therapy fails or when complications occur.

## METHOD

This study consisted of a report on our institutional experience with revisional bariatric surgery and a systematic review of the literature based on reference analyses retrieved from current databases such as PubMed, Lilacs and SciELO. The search strategy was defined by terms related to [bariatric surgery] in combination with [revisional surgery] as well as [surgery complications] in English, Portuguese or Spanish language. 

## RESULTS

### Rationale of revisional bariatric surgery

The success of bariatric surgery is often defined by the achieved weight loss caused by the% EWL (excess weight loss). It can be specified by:

% EWL = (preoperative body weight - current body weight)/(preoperative body weight - ideal body weight) x 100

A successful bariatric surgery was defined by Brolin et al. with a %EWL≥0%^8^. Furthermore, the achieved weight loss was classified by Reinhold criteria^9^, been modified by Christou et al. 2006, and now find a wide clinical application^10^:a) incompetent weight loss if BMI>35 kg/m^2^; b) good weight loss when BMI=30 to 35 kg/m^2^; c) excellent weight loss if BMI<30 kg/m^2^

Current guidelines or consensus items of the respective professional societies for performing bariatric conversion procedures are not available. However, this can be indicated in clinical practice by the following characteristics: a) incompetent weight loss (BMI>35 kg/m^2)^; b) rebound weight gain (BMI>35 kg/m^2)^; and c) recurrence of a metabolic disorder or complication of the initial process (acute and chronic).Before a possible conversion engaging conservative treatment methods is essential. Furthermore, they should be performed over a longer period, in general longer as two years, before the indication for surgical revision is made. Current guidelines for the timing of the implementation of a conversion procedure are not well delineated^11,12^ so that restraints of the health insurance companies may occur.

Audit procedures were performed in accordance with the German Bariatric Surgery Registry (GBSR, prospective quality assurance study for the surgical treatment of obesity at the Institute of Operational Medicine at Otto von Guericke University, Magdeburg) in 8.6% of cases^11^. In 7% conversion operations were carried out^11^, the lack of weight loss or weight regain were the main reasons^13^.

### Conversion of sleeve gastrectomy to RYGB

LSG is performed more frequently in recent years. However, few long-term data show often insufficient weight reduction^3^ ^,^ ^14^ ^,^ ^15^. The initial weight loss after LSG is given as over 50% (EWL), but it takes up to 20% of cases of regaining weight^16^ ^,^ ^17^. In addition in a LSG, symptoms of gastroesophageal reflux may occur and are usually refractory to medical therapy^18^. In both cases, a conversion process can be performed^19^. The conversion of a LSG to a RYGB is the method of choice and reliably causes a new weight reduction at a slightly increased rate of complications^20^ ^-^ ^22^. The mortality of the conversion to RYGB is less than 1%^21^ ^,^ ^23^.

#### Technical aspects

**FIGURE 1:**
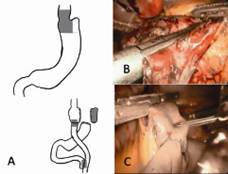
Conversion from laparoscopic sleeve gastrectomy (LSG) to RYGB: A) schematic illustration of the conversion where LSG is cut horizontally with a linear stapler, preserving the blood vessels and, in some cases, reduction of the pouch is indicated, for dilated sleeves; B) linear stapler is set horizontally to delineate the pouch formal RYGB is performed; C) the afferent loop of the performed gastrojejunal anastomosis is cut by a linear stapler to form the Y-Roux.

The conversion to RYGB can be performed laparoscopically. In most cases, an extended adhesiolysis between the stomach and liver is necessary. After retraction of the liver, the omentum minus is dissected and the stomach tube mobilized toward diaphragm. The stomach tube is divided horizontally by a stapler (Figure 1). The vessels and nerves along the lesser curvature are preserved, so that the blood flow to the rest of the stomach remains and maintaining pylorus function. In cases in which the gastric tube is already dilated, a further reduction of the pouch is necessary. Another stapler cartridge truck (60 cm) is placed vertically in order to reduce the new pouch laterally. Resection of the remaining excluded stomach is not usually necessary, except when the blood support or vagus nerve are damaged.^24^. After this, the completion of RYGB is carried out in a tipical way.

### Conversion of gastric band to RYGB 

The laparoscopic adjustable gastric band shows in the initial period low perioperative morbidity^25^. In long term, however, increased complication rates including gastric perforation, band dislodgement, band migration, gastroesophageal reflux disease or endoluminal gastric band erosion are described^26^ ^-^ ^28^. Endoscopic gastrointestinal band extraction is preferable than minimally invasive or surgical extraction^29^. However, this engagement is not always feasible and technically demanding. Nevertheless, inadequate weight loss after a gastric band is the most common cause to perform a conversion to LSG or RYGB^30^. The conversion of the laparoscopic adjustable gastric band for RYGB shows good clinical results^10^ ^,^ ^33^. The weight loss and permanent weight reduction is comparable to the primary RYGB, although the indication is provided due to insufficient weight reduction or due to gastric banding associated complication. The largest study of the conversion from the gastric band to RYGB (n=642) had a mortality of 0%^34^. Complication rate was 9.7%, with 3.6% of patients having serious complications. The long-term results (observation period of 10 years) are similar to the results of a primary RYGB. The conversion of the gastric band to RYGB can be done one-stage or two-stage. In the two-stage conversion the first step is the removal of the band and subcutaneous port. In a second procedure the gastric bypass is applied. In this regard, the data are controversial and neither of the two approaches has a clear advantage on. The advantages of the one-stage process, the reduced operative time, length of hospital stay and economic factors are given. In addition, the gastric band can be used for orientation of the pouch construction. In the two-stage process after band removal there is a waiting period of 2-6 months to apply the gastric bypass as a second step. In summary, similar complication rates are listed, even though the evidence is very inhomogeneous and no final statement can be made in systematic reviews of both methods^10^ ^,^ ^33^. Moon et al. showed that the conversion of a gastric band to sleeve gastrectomy or RYGB comprises comparable results regarding weight loss and the rate of complications^35^.

#### Technical aspects

**FIGURE 2:**
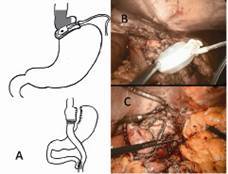
Conversion of a laparoscopic adjustable gastric band to RYGB: A) schematic illustration of the conversion where the linear stapler is set proximally to the scarred tissue of the former band, when possible; B) adhesions and scars to gastric wall are solved; C) the pouch is performed through a horizontal and a vertical stapler, and formal RYGB is performed.

The patient can be explored laparoscopically and adhesions (especially between the stomach and liver) are dissected. The band tube is separated from the gastric part of the band and extracted later with the port through a trocar. The gastric band and the surrounding capsule are dissected with scissors or the ultrasonic scissors. Before the actual conversion is done, endoscopy is performed to rule out a possible erosion of the gastric band. In the event of a defect, the elective conversion must be stopped and postponed until the complete healing. The construction of a pouch (Figure 2), avoids the scarring tissue and preferable distal to the former gastric band. The tissue in the area of ​​the former gastric band is usually very vulnerable and not suitable for anastomosis. Following, the other regular steps of RYGB are performed.

### Conversion of vertical banded gastroplasty (Mason) to RYGB

The vertical gastroplasty in 1982, described by Mason^31^, was for years the bariatric method of choice^32^. Initial weight loss is mostly successful^33^ although the patients may develop complications and have in the long term insufficient weight reduction^12^. The surgical revision is often combined with conversion to bariatric procedures, being to RYGB the most common. Mortality is related to be less than 1%^34^ ^,^ ^35^. The complication rates occur at a frequency of 9-19%^34^ ^-^ ^37^. Patients with a vertical banded gastroplasty, which must be revised surgically without carrying out a conversion to RYGB, have disappointing results and will be revised again in 50% of cases. In contrast, there is a significantly lower rate of re-revisions (under 5%), if simultaneously RYGB conversion is performed as the first revisional procedure^34^ ^,^ ^38^ ^,^ ^39^. Overall, the conversion of vertical gastroplasty to RYGB is useful, although it is associated per se with increased mortality and is inferior to other bariatric procedures.

#### Technical aspects

**FIGURE 3:**
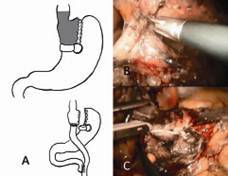
Conversion from vertical stapled gastroplasty (Mason) to Roux-en-Y-gastric bypass: A) schematic illustration of the conversion with the stapling line to perform the pouch is horizontally set, without crossing the previous vertical gastroplasty; B) adhesions in the region of the gastroplasty are solved, to identify the ring, a 45 mm stapler is set horizontally and a further stapler (60 mm) is also set vertically (medial or at the Mason gastroplasty) to allow distal flow, if the former pouch is not resected; the ring can be or not extracted, depending on the position of the new pouch; C) construction of the pouch using 45 stapler and the pouch is anastomosed to the alimentary limb, and a formal RYGB is performed.

The patient is submitted to an exploratory laparoscopy and adhesions is solved (especially between the stomach and liver). The left lobe is fixed by a retractor. The pouch region is dissected. A linear stapler (45 mm) is set horizontally without crossing the vertical line of the former vertical gastroplasty (Figure 3). The band is often not removed and remains in the existing position in the patient^40^. Another stapler (60 mm) is placed medial of the vertical gastroplasty to ensure an outflow of the old pouch distally. Another option is the resection of the ring and use the previous vertical staple line as the new pouch. All staple lines can be oversewn with absorbable suture. Following, a standard RYGB is typically performed.

### Conversion of RYGB to jejunal interposition with resection of excluded stomach and alimentary limb (Branco switch)

In a few cases, after creating a RYGB, severe hypoglycaemia with neuroglycopenia (NIHHPB - non-insulinoma hyperinsulinemic hypoglycaemia post-bypass) can occur. The prevalence in the literature shows a high variety and is in some study about 1%^41^ ^,^ ^42^. Recently published study (n=1206) showed cumulative 5-year prevalence of 13.3%^43^. Patients were included only without preoperative diabetes before the original operation and were classified according to the Edinburgh hypoglycemia scale. One reason for variety in the occurrence in different databases is attributed to an insufficient medical detection of hypoglycemic episodes.

Patients with severe hypoglycemia are hospitalized and need to receive adequate diagnosis^44^. This includes a detailed medical history (temporal relationship between symptoms and food intake) including the detection of the current medication (interference with beta-blockers, ACE inhibitors, antibiotics, angiotensin receptor blockers). Furthermore, hormone levels (cortisol, somatropin, insulin-like growth factor, thyroid hormones), heart, kidney and liver function, should be checked which can cause hypoglycemia. Functional mixed-meal tolerance test^44^ is preferable to the oral glucose tolerance test, but it is difficult to compare among series. For the possibility of insulinoma, neuroendocrine tumors imaging is recommended. In individual cases, hyperplastic beta cells can be selectively stimulated and localized by calcium gluconate^45^.

The primary recommended treatment is conservative, with dietetic measures and/ r medication. A few patients do not respond to these therapies, and can optionally be treated surgically. Various methods are reported in the literature, although all have a very small number of cases. The restoration of the RYGB anatomy through a gastrogastrostomy including the resection of the alimentary loop^46^ or even the placement of a gastrostomy in the distal stomach, could treat the NIHHPB in selective cases^47^. The mechanism of such interventions is the hypothesis that the transport of food through the alimentary loop excluding the duodenum is responsible for NIHHPB. Furthermore, a so-called nesidioblastosis - hypertrophy of beta-Langerhans cells of the pancreas in adults - is presumed to be installed in the pancreas, which is associated with an autonomous insulin secretion. The exact pathological mechanism is not fully understood^48^. In severe cases with refractory neuroglycopenia episodes and severe hypoglycemia, partial or total pancreatic resection have been considered^49^ ^,^ ^50^. Alternatively, the occurrence of NIHHPB can currently be treated by a Branco switch procedure. The technique was developed by the group of Alcides Branco, from Curitiba, Brazil^51^. The goal of the procedure is to restore the duodenal continuity, resection of most of the alimentary limb and excluded stomach, so that the passage of food through the duodenum allows for reduction of delayed insulin and pancreatic secretion. By still maintaining malabsortion and restriction, the desired weight reduction is still guaranteed.

So far, nine patients in our study have been laparoscopically operated by the Branco switch technique due to severe hypoglycemic episodes^51^. Postoperative normal pre- and postprandial glucose and insulin levels were measured. Hypoglycemic episodes stopped in all patients and were therefore treated successfully. The BMI was 32.5 kg/m^2^ before and 29.9 kg/m^2^ six months after the Branco switch. Ulcers, dumping and other complications have not occurred in the mean observation period of two years.

The medical evidence is low in endoscopic and laparoscopic procedures for treatment of NIHHPB that are conducted in individual specialized centers. The TORE procedure (transoral endoscopic outlet repair of G-J anastomosis), by reducing the diameter of the gastroenteroanastomosis, is showing good results for treatment of dumping syndrome and weight regain after RYGB.

#### Technical aspects

**FIGURE 4:**
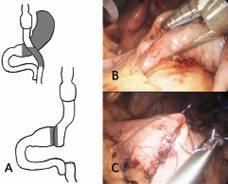
Conversion from RYGB to jejunal interposition with subtotal gastrectomy - Branco switch: A) schematic illustration of the conversion for therapy of severe hypoglycemia after RYGB; the primary objective is transforming the RYGB and restoring the continuity to the duodenum through a jejunal interposition and subtotal gastrectomy; B) performing a pylorus-preserving gastrectomy at 2-4 cm of remaining antrum and, finally, the alimentary limb is resected at 20 cm from the gastrojejunal anastomosis till the Y-Roux anastomosis; C) the 20 cm remaining alimentary limb becomes a handsewn anastomosis to the rest of the antrum and the Y-Roux is closed by a stapler to finalize the resection of the alimentary limb.

The conversion procedure can be performed laparoscopically. Mobilization of the stomach is done and the antrum is stapled close to the pylorus (2-4 cm) to perform partial gastrectomy. The alimentary jejunal loop is resected until 20cm to the G-J anastomosis, which remains intact. The 20 cm length alimentary jejunum is then anastomosed to the antrum close to the pylorus. The gastrojejunal anastomosis is performed handsewn (Figure 4). The former Y-anastomose is also closed (resection of the alimentary limb) by a staple. A total of four or five trocars are set to RYGB conversion to Branco switch. A novel vacuum liver retractor can be used to avoid an additional trocar^52^.

In general a larger diameter should be used (4.2 mm) because the gastric wall can be thickened by scarring in the use of stapling devices. Due to the increased complication rate of revisional surgery, the use of large stapler charges (black or green) and intra-abdominal drainage is recommended^11^.

### Endoscopic revisional procedures

Apollo transoral reduction of gastrojejunal anastomose (Trans oral outlet reduction/TORE)

**FIGURE 5:**
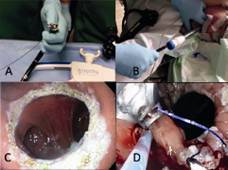
Endoscopic revision of RYGB: A) the transoral reduction of the gastrojejunal anastomose after RYGB (transoral outlet reduction/TORE) is performed endoscopically, using a special full-thickness suture device (Apollo Overstich(r), Austin, TX, USA); B) a special overtube is inserted to prevent esophageal damage; C) the enlarged gastrojejunal anastomosis (around 5 cm) is prepared for sutures using Argon beamer; D) the dilated outlet anastomosis is effectively reduced to less than 9 mm through Overstich(r) sutures.

Weight regain occurs up to 20% of cases after RYGB^53^ ^,^ ^54^. For these patients, novel endoscopic methods can be used to reduce the weight. The transoral reduction in the diameter of gastrojejunal anastomosis (TORE) is performed endoscopically. A special suture device (Apollo Overstich(r), Austin, TX, USA) is a full-thickness endoscopic suture, laterally set so that the diameter and thus the output of the distal stomach pouch is reduced (Figure 5)^55^ ^,^ ^56^. The method has a restrictive effect on food intake and can cause renewed weight loss in recent series and in our experience. The pre-interventional evaluation is important to evaluate whether the gastric pouch or the gastrojejunostomy are dilated, as only with enlarged diameter the use of TORE technique is indicated. The patient can be discharged in the early post-interventional period (<24 h) after a short monitoring. In the largest prospective study (n=150) a reduction in the EWL of 19% was achieved after an observation period of three years^57^. No severe complications occurred, although abdominal pain (4%), hematemesis/melena (3.3%) and nausea (2%) were observed. The diameter of the gastrojejunal anastomosis was reduced from a mean of 24 mm to 9 mm (p<0.05).

### Apollo endoscopic sleeve gastroplasty/ESG

**FIGURE 6:**
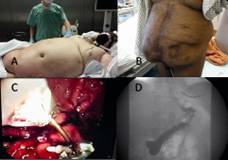
Endoscopic sleeve gastroplasty (ESG): A) novel indications for primary endoscopic sleeve gastroplication, as a first step in high risk patients for preparation for a surgical second step in 9-12 months; BMI=72 (315 kg) with respiratory insufficiency and tracheostomy; B) as a primary procedure in patients with giant hernia and adhesions; in this patient, laparoscopic SG was aborted due to adhesions, and ESG was indicated; C) the performance of ESG as a primary bariatric procedure is set through consecutive full-thickness sutures from the incisura angularis to proximal; the sutures are interrupted, so the gastric secretion and food can have free transit between the gastroplasty; D) postinterventional radiologic study after ESG.

The endoscopic system for producing sleeve intragastric plication without the need for an abdominal incision was first published in 2013^60^. This intervention is indicated for primary indication for morbid obesity, but this can also be used for the revision of a LSG. Here, a full-thickness suture is performed with a special suture device (Apollo Overstich) using a single or double row suture vertically placed along the greater curvature. The suture is set interrupted, so that the secretion of the stomach can completely reach the duodenum. The first documented clinical series in Germany was carried out by our group at the Charité, Virchow Clinic, Berlin. The first patient had a BMI of 45 kg/m^2^ and a huge fat apron and a giant incisional hernia due to multiple abdominal surgeries (n=14). Two attempts of laparoscopic and open bariatric operation were aborted due to adhesions. The procedure took a total of 90 min with no complications (Figure 6)^61^. After six months was observed a weight reduction of 25% (BMI=30.2 kg/m^2)^. Further series in our institution are studying the indication of endosleeve as a first step for patients super-super obese (BMI over 60 kg/m^2)^ and for high risk patients with cardiovascular impairment as a definitive procedure, and for renal transplant recipients with morbid obesity as a preparation for the kidney transplant.

The available published studies show very good results, but the number of patients is still low^62^ ^,^ ^63^. In the longest follow-up study (n=25, observation period one year) the body weight was reduced in 19% and the EWL in 54%^64^. No complications were documented. In comparison to LSG, the gastric blood supply and innervation are preserved. The process can be reversible in the first two weeks. It is possible that ESG can be indicated for very high BMI in the concept of two step procedure instead of LSG, as performed in our first series in Charité, Berlin. The technique can also be indicated after an initial reduction in weight and applied secondarily during RYGB^24^ ^,^ ^65^.

Endoscopic Apollo revision after LSG can be also possible for treatment failure, that occurs in up to 20% of cases due to insufficient weight loss or weight regain^16^ ^,^ ^17^. If large diameter of the sleeve is diagnosed by gastric radiologic volumetry, a so-called endoscopic re-sleeving can be carried out^58^ ^,^ ^59^. The literature for endoscopic re-sleeving a failed LSG is still scarce. 

## CONCLUSION

The growing incidence of bariatric surgery is associated with an increase in the importance in revisional techniques. Inadequate weight loss or weight regain are the most common indication for revision and conversion. Preliminary results justify the use of RYGB as conversion procedure of choice. Alternatively, endoscopic procedures with low risk can be used with less morbidity and mortality, although long-term results are still not available.
